# Biological crystallography: new methods, new challenges

**DOI:** 10.1107/S2052252515003541

**Published:** 2015-02-26

**Authors:** Edward N. Baker

**Affiliations:** a School of Biological Sciences, University of Auckland, Private Bag 92-019, Auckland, New Zealand

**Keywords:** biological crystallography, free electron lasers, editorial, IUCrJ

## Abstract

The ability of crystallography to illuminate biology and contribute to medical advances continues to grow. Recent advances expand its reach while also asking questions of its practitioners.

A highlight of the International Year of Crystallography, which we celebrated last year, was the 23rd Congress of the International Union of Crystallography, held in Montreal. Among the many microsymposia, covering the extraordinary breadth of modern crystallography and its applications, was one entitled ‘The beginnings of biological crystallography’. Somewhat to the surprise of those involved, this drew a huge audience, possibly the largest for any session at the entire Congress. What I think this reflected was both pride and wonder at how we have reached the point we have today. However, the talks also reminded us that most of the things that people worried about then are still alive as challenges today. Despite the huge advances over recent years, the fundamentals of crystallography remain the same. There are big differences, of course: crystals had to be very large, data collection took months, if not years, phase determination was limited to isomorphous replacement and coordinates were derived from wire models built by hand. In fact, the diffraction data were usually very good because so much attention was paid to quality, phases were over-determined and the resulting structures had few errors because we worried about the fit of every residue.

This journal highlights the very best of new research across the whole breadth of crystallography; methods, ideas, results. In the biological sphere some outstanding papers have addressed a broad range of topics: the assembly of SNARE complexes that orchestrate cellular trafficking; new approaches to inhibition of a promising *Leishmania* drug target; the complete structure of a complex that synthesizes *S*-adenosylmethionine; pressure effects on biological membranes; advances in the use of small-angle scattering to enhance biological insights; the *PDB_REDO* server for optimizing models; and many others.

We have also seen some spectacular methodological advances, notably in the applications of free electron lasers (FELs) to biological crystallography. These have enabled the assembly of complete data sets from hundreds of thousands of femtosecond images taken from tiny crystals as they are flowed in a liquid suspension across the X-ray beam. This capability has led to the new concept of serial crystallography (Chapman *et al.*, 2011 ▶). Now, in articles published in **IUCrJ**, it has been shown that similar ideas can be applied using microfocus beamlines at conventional third-generation synchrotrons (Stellato *et al.*, 2014 ▶). In one example, the structure of an enzyme, procathepsin B, was determined from 28 800 diffraction images taken from a cryo-cooled suspension of some 5000 **in vivo** grown microcrystals (Gati *et al.*, 2014 ▶). In another, the 2.4 Å structure of a membrane protein was determined by serial millisecond crystallography, from over 1 million images taken as randomly oriented, lipidic cubic phase crystals were flowed through the beam (Nogly *et al.*, 2015 ▶).

Interestingly this approach has several features that hark back to the early days of macromolecular crystallography. Using a liquid suspension of crystals, data can be collected at room temperature, a distinct advantage for those proteins whose crystals cannot be frozen without loss of crystallinity. Moreover, as Michael Rossmann pointed out in a recent commentary (Rossmann, 2014 ▶), the merging of data collected from many randomly oriented crystals tends to average out their varying absorption profiles – a significant factor in data quality and one that is often overlooked today. It is yet to be seen how widely the serial crystallography approach will be adopted, and how it will deal with fundamental steps such as space group determination in cases where crystals are not already characterized. However, it has exciting advantages: thousands of crystals can be screened, and data sets assembled, in a very small time and with less than 1 mg of protein. The fast timescale is also well suited to time-resolved studies.

One final editorial comment. Crystallography is a wonderful discipline that gives unparalleled insights into the natural world. It is data-rich, and increasingly relies on powerful, automated software at all steps of structure determination. Practitioners must, however, be ever more vigilant to avoid errors as they become more removed from ‘hands-on’ crystallography. And bioinformatics resources such as those described by Berman *et al.* (2015 ▶) in this journal have become critical for transforming data into knowledge and true biological insight.

A full listing of the biology and medicine papers published in **IUCrJ** can be found at http://journals.iucr.org/m/services/biol_med.html.
